# Being moved as one of the major aesthetic emotional states: A commentary on “Being moved: linguistic representation and conceptual structure”

**DOI:** 10.3389/fpsyg.2015.00343

**Published:** 2015-03-31

**Authors:** Vladimir J. Konečni

**Affiliations:** Department of Psychology, University of California, San DiegoSan Diego, CA, USA

**Keywords:** being moved, aesthetic trinity theory, aesthetic awe, thrills or chills, peak aesthetic experiences, forgiveness, generosity, self-sacrifice

Recently in this journal, Kuehnast et al. (2014) empirically explored the semantic, psychological, and emotional features of *being moved*. The purpose of this commentary is to relate the results and discussion of Kuehnast et al. to aesthetic concepts and theory. One of the key tasks of both psychological and philosophical aesthetics is the analysis of peak experiences in response to artworks and architectural objects (both broadly defined), as well as natural wonders (e.g., Kant, 1790/1914; Panzarella, 1980). In the recently proposed Aesthetic Trinity Theory (Koneèni, 2005; Konečni, 2011; ATT hereafter), a tripartite hierarchy is delineated: Aesthetic Awe (AeA), a rare, intense, highly memorable peak experience; the state of Being Moved (BM), a more common and less pronounced experience; and (physiological) Thrills or Chills (ThCh), the most frequently experienced and observed response.

The theoretical expectation is that AeA is most reliably induced by a sublime stimulus-in-context. The sublime is, of course, a classical notion in philosophy (Koneèni, 2005; Konečni, 2011). Unlike some ambivalent prior positions, it is considered in ATT to be external to the observer; it is defined, independently of AeA, as a complex stimulus located in a unique setting and characterized by great rarity, beauty, physical grandeur, and, often, inaccessibility. In contrast to many instantiations of non-aesthetic awe (Keltner and Haidt, 2003), ATT stipulates that the experiencer must have existential security for AeA to occur (Koneèni, 2005). The pyramids of Giza are the prototypical sublime stimuli. A certain amount of preliminary empirical work on the sublime stimuli has been carried out (Konečni et al., 2007, Experiment 3).

In contrast, since the pioneering work of Goldstein (1980) and Panksepp (1995), the comparative ease of experimental control and reliable measurement have made ThCh an increasingly popular research topic—mostly as a response to music (e.g., Benedek and Kaernbach, 2011). It is, however, of interest that whereas there is ample anecdotal and literary evidence that the experiences of AeA may be life-changing events, a series of laboratory experiments by Konečni et al. (2007) failed to demonstrate any effect of music-induced ThCh on participants' mood, self-concept, and altruism (willingness to donate blood). In a study that used positron emission tomography, Blood and Zatorre (2001) found that the occurrence of music-induced ThCh is correlated with activity in brain regions implicated in reward and emotion (ventral striatum, midbrain, amygdala, etc.). However, the procedure used leaves open the question of which aesthetic response was induced. Participanta selected their own ThCh-inducing music and one participant's favorite music was another's control music: no participant responded to control stimuli with ThCh. These results strongly suggest that all music stimuli acquired their within-subject ThCh-inducing ability by being associated with various situational and personal factors on prior listening occasions. Therefore, a distinct possibility is that in the experiment, participants experienced not only ThCh (as confirmed by the psychophysiological measures), but also the more intense and rare BM.

Participants in the Kuehnast et al. (2014) study responded to primes (the present and past participles of eight verbs, including “moving” and “moved”) on a free word-association task. With regard to the semantic field and conceptual structure of BM episodes, the main elicitors were (a) significant life events and (b) art stimuli, especially film and music, which provides empirical support for the theoretical proposals from ATT (Konečni, 2011). However, literary examples were not salient, which may be due to task attributes or the general decline of reading culture. Historically, innumerable authors have moved readers by describing the same significant life events that Kuehnast et al. detected, but, in addition, novels are replete with characters who are moved by such events, as well as characters who are moved by other characters being moved (Koneèni, 2005). The word association task also failed to reveal other important elicitors, such as acts of generosity, forgiveness, and, especially, self-sacrifice, which have been found to induce ThCh in the study by Konečni et al. (2007, Experiment 2: both self-sacrifice leading to a happy end and one failing to achieve it induced more ThCh than the control, especially the latter).

In complete agreement with ATT, Kuehnast et al. proposed that BM is a “discrete emotional state having a unique quality” (p. 8; cf. Cova and Deonna, 2014) and identified joy and sadness as the main emotional ingredients of BM. These considerations highlight an important aspect of ATT—the position that AeA is a primordial mixture of joy and fear, and that it is related to the fundamental emotions without itself being one (Koneèni, 2005). The latter proposal is also addressed by Kuehnast et al. (2014), for they suggest that “‘movingness’; is not by definition included in the concepts of joy and/or sadness, but rather represents an additional feature found in highly select episodes of sadness and joy” (p. 8).

It would seem that the theoretical work on ATT (Koneèni, 2005; Konečni, 2011; Konečni et al., 2007) and the recent empirical work of Kuehnast et al. (2014) jointly provide a solid basis for future work on emotion-related peak aesthetic experiences.

## Conflict of interest statement

The author declares that the research was conducted in the absence of any commercial or financial relationships that could be construed as a potential conflict of interest.
